# Efficacy of Brain-Computer Interface Therapy for Upper Limb Rehabilitation in Chronic Stroke: Systematic Review and Meta-Analysis of Randomized Controlled Trials

**DOI:** 10.2196/79132

**Published:** 2026-01-28

**Authors:** HongJie Chen, GuoJun Yun

**Affiliations:** 1 Department of Rehabilitation Medicine Shenzhen Children's Hospital Shenzhen City, Guangdong Province China; 2 College of Rehabilitation Medicine Jiamusi University Jiamusi, Heilongjiang China

**Keywords:** brain-computer interface, chronic disease, upper limb function, stroke, rehabilitation, meta-analysis

## Abstract

**Background:**

Over 50% of people with chronic stroke experience persistent upper limb dysfunction. Brain-computer interface (BCI) therapy, creating a sensorimotor loop via neural feedback, is a promising alternative; yet, its optimal application remains unclear.

**Objective:**

This meta-analysis evaluates BCI’s efficacy on motor function, tone, and activities of daily living (ADL) in chronic stroke and identifies optimal feedback modalities and intervention parameters.

**Methods:**

We systematically searched Cochrane Library, Embase, PubMed, Scopus, Web of Science, and Wanfang Data from inception to October 2025 for randomized controlled trials (RCTs) comparing BCI-based training to control interventions in adults with chronic stroke. Primary outcomes were upper limb motor function (Fugl-Meyer Assessment for upper extremity [FMA-UE], Action Research Arm Test [ARAT]), muscle tone (Modified Ashworth Scale [MAS]), and ADL (Modified Barthel Index [MBI], Motor Activity Log [MAL]). Screening, data extraction, and risk-of-bias assessment were performed independently. Meta-analysis used a random-effects model with Hartung-Knapp-Sidik-Jonkman adjustment. Pooled mean differences (MDs) with 95% CIs and 95% prediction intervals (PIs) were calculated. Subgroup analyses examined feedback modalities, intervention intensity, and follow-up effects. Sensitivity analysis was also conducted.

**Results:**

From 3529 records, 21 RCTs (650 participants) were included. BCI training significantly improved motor function (FMA-UE: MD 2.50, 95% CI 0.60-4.40; *P*=.01; 95% PI –2.52 to 7.22) and ADL performance (MBI: MD 8.38, 95% CI 2.23-14.53; *P*=.02; 95% PI –3.92 to 20.53; MAL: MD 2.09, 95% CI 0.42-3.76; *P*=.03; 95% PI –0.69 to 4.54). No significant effects were observed for fine motor skills (ARAT: MD 0.18, 95% CI –0.27 to 0.62; *P*=.30; 95% PI –3.64 to 3.99) or muscle tone (MAS: MD –0.48, 95% CI –1 to 0.03; *P*=.06; 95% PI –1.27 to 0.35). Subgroup analyses revealed that BCI-functional electrical stimulation (FES) yielded the greatest improvement in motor recovery (FMA-UE: MD 5, 95% CI 1.86-8.13; *P*=.01). The optimal intervention protocol was identified as 30-minute sessions, administered 4-5 times per week over 2 weeks (total of 10-12 sessions). However, benefits were not sustained at follow-up.

**Conclusions:**

Low- to moderate-certainty evidence suggests that BCI training, particularly the BCI-FES paradigm, can improve upper limb motor function and ADL in people with chronic stroke on average. However, wide prediction intervals indicate the effect may vary substantially across settings, ranging from negligible to beneficial. Subgroup analyses suggested a potential optimal protocol of 30-minute sessions, 4-5 times per week for 2 weeks, but these findings are limited by the small number of studies in each subgroup and the high risk of bias in several included trials. Therefore, this proposed protocol should be viewed as preliminary and requires validation in future, high-quality RCTs. Future research should also focus on identifying patient subgroups most likely to benefit and on strategies to sustain long-term gains.

**Trial Registration:**

PROSPERO CRD420251063808; https://www.crd.york.ac.uk/PROSPERO/view/CRD420251063808

## Introduction

Stroke, a neurological disorder resulting from cerebrovascular rupture or obstruction, leads to motor, speech, and cognitive impairments, consequently compromising performance in activities of daily living (ADL) [1]. Upper limb motor dysfunction represents one of the most prevalent sequelae of stroke [2,3], affecting a substantial proportion of survivors. Contemporary evidence indicates conventional rehabilitation strategies achieve optimal therapeutic gains predominantly within the first 6 months poststroke [4,5]. However, a substantial proportion of patients miss this critical window. For those in the chronic phase, current interventions demonstrate limited efficacy [6]. Notably, comparative studies reveal that stand-alone exoskeleton-assisted training or functional electrical stimulation (FES) provides comparable therapeutic benefits to conventional rehabilitation in chronic stroke cohorts [7]. Given the persistent, unmet rehabilitation needs in this population, brain-computer interface (BCI)–based training merits serious consideration. By enabling heightened patient engagement in volitional motor tasks [8], BCI represents a paradigm-shifting approach with significant potential to enhance recovery trajectories.

BCI technology establishes a direct communication pathway between the brain and an external device, bypassing damaged neural pathways. Fundamentally, BCI systems acquire and decode characteristic patterns of neural activity associated with user intent, such as motor imagery [9]. These decoded signals are then translated into commands to operate external feedback devices. BCI training represents an emerging neurorehabilitation technology based on this principle. This approach collects and decodes characteristic brain activity patterns, translating them into computerized commands to operate external feedback devices. These devices include FES [10], robotic exoskeletons [11], and visual feedback systems [12]. BCI systems are broadly categorized as invasive or noninvasive based on signal acquisition methodology. Due to safety concerns and practical limitations associated with invasive techniques [13], noninvasive BCIs are predominantly favored in current rehabilitation practice [14]. Primary noninvasive signal acquisition modalities encompass electroencephalography (EEG), magnetoencephalography, functional near-infrared spectroscopy (fNIRS), and functional magnetic resonance imaging. Among these, EEG stands as the predominant modality for signal acquisition in clinical rehabilitation settings [15]. The level of control exerted over external devices is contingent upon the specific neural signal source used. Integrating diverse neural signals within BCI frameworks enables more refined and efficient operation of feedback apparatus [16]. In contrast to conventional rehabilitation methods, the BCI paradigm establishes a “central-peripheral-central” closed-loop model. This approach holds promise for facilitating more timely movement adjustments and compensation strategies in people who have experienced a stroke [17], potentially offering greater alignment with personalized rehabilitation requirements.

Since the initial report on EEG-based BCI training in 2009 [18], numerous studies have demonstrated the efficacy of BCI interventions for improving upper limb motor outcomes in people who have experienced a stroke, including muscle strength, motor function, and ADL [12,19,20]. While some meta-analyses have addressed stroke stages in subgroup analyses, recent systematic reviews and meta-analyses primarily focus on comparing the magnitude of improvement across different stroke stages for a single primary outcome measure. For instance, subgroup analyses in studies by Xie et al [21] and Yang et al [22] solely compared BCI efficacy on upper limb function across stroke stages, without specifically evaluating the long-term therapeutic effects of BCI in people with chronic stroke. Furthermore, the influence of critical clinical parameters—such as intervention duration and session frequency—remains inadequately examined. A systematic analysis of BCI-based training protocols optimized for chronic stroke populations has yet to be conducted. Addressing these aspects is crucial for developing tailored rehabilitation protocols to enhance upper limb recovery, ADL performance, and overall quality of life in people with chronic stroke.

Therefore, this meta-analysis was conducted with three specific aims:

1. To systematically evaluate the efficacy of BCI-based training on upper limb motor function, muscle tone, and ADL exclusively in people with chronic stroke;
2. To perform in-depth subgroup analyses of key moderating factors—including BCI feedback modalities, intervention intensity parameters (session duration, frequency, and total sessions), and follow-up effects—which have been inadequately addressed in prior syntheses;
3. To attempt to propose optimized BCI intervention protocols tailored to the chronic stroke population based on current evidence.

By addressing these gaps, this review seeks to provide clearer guidance for clinical practice and future research in BCI-based stroke rehabilitation.

## Methods

### Protocol and Registration

This systematic review was registered with PROSPERO (International Prospective Register of Systematic Reviews), the international systematic review database, bearing identifier CRD420251063808. This meta-analysis followed the PRISMA (Preferred Reporting Items for Systematic Reviews and Meta-Analyses) guidelines published in 2020 [23] (Multimedia Appendix 1).

### Search Strategy

The systematic literature search was designed, conducted, and reported in accordance with the PRISMA-S (Preferred Reporting Items for Systematic Reviews and Meta-Analyses – Search Extension) guideline [24]. An experienced information specialist (HJC) developed the search strategy in collaboration with the review team.

We executed a comprehensive search across 6 electronic databases from their inception until October 16, 2025, including PubMed (via the National Library of Medicine), Embase (via Ovid), Cochrane Central Register of Controlled Trials (via Wiley), Scopus (via Elsevier), Web of Science Core Collection (via Clarivate Analytics), and Wanfang Data. These platforms were selected to ensure extensive coverage of both international and Chinese literature. All databases were searched individually; no multidatabase searching was performed on a single platform.

The search strategy used a combination of controlled vocabulary (eg, MeSH [Medical Subject Headings] in PubMed and Emtree in Embase) and keywords related to the core concepts of “brain-computer interfaces,” “stroke,” “upper extremity,” and “rehabilitation.” The complete search strategies for all databases are provided in Multimedia Appendix 2. To maximize sensitivity, no restrictions were placed on language, publication date, or study design during the search. Similarly, no published search filters were used, and the strategy was developed de novo for this review, not adapted from prior work.

To enhance the robustness of the search, the PubMed strategy underwent peer review by an information specialist prior to execution, following the Peer Review of Electronic Search Strategies (PRESS) guideline framework [25]. Additionally, in attempts to obtain missing or incomplete data, we contacted the corresponding authors of studies via email.

Beyond the methods above, we did not systematically search study registries, websites, or gray literature, nor did we use citation searching.

The total number of records retrieved from each database is documented in the PRISMA flow diagram. All identified records were imported into EndNote X9 (Clarivate) for management, where duplicates were removed using the software’s automated deduplication feature, followed by a manual verification process conducted independently by 2 reviewers (HJC and GJY).

Inclusion criteria comprised (1) population: adults (>18 years) diagnosed with chronic stroke (>6 months poststroke), exhibiting stable vital signs and alert consciousness; (2) intervention: receiving any form of BCI-based training; (3) control: receiving either sham BCI interventions or conventional rehabilitation therapy (eg, physical therapy, occupational therapy, and treadmill training); (4) outcomes: assessment using the Fugl-Meyer Assessment for upper extremity (FMA-UE), Action Research Arm Test (ARAT), Modified Ashworth Scale (MAS), Modified Barthel Index (MBI), and Motor Activity Log (MAL); (5) study design: randomized controlled trials (RCTs) published in English or Chinese.

Exclusion criteria were (1) nonprimary research publications (eg, reviews, meta-analyses, systematic reviews, and conference abstracts); (2) studies reporting outcome measures inconsistent with those prespecified for analysis; and (3) publications with incomplete or irretrievable data.

### Selection Process

All records identified through the database searching were imported into EndNote X9 (Clarivate Analytics) for management. The total number of records retrieved from each database and information source was documented. Duplicates were removed using a 2-step process: first, automatically using EndNote’s built-in deduplication feature, followed by a manual verification conducted independently by 2 investigators (HJC and GJY). The screening process was then carried out in 2 stages; first, titles and abstracts were screened against the inclusion criteria; second, the full texts of potentially eligible records were retrieved and assessed. Both investigators independently evaluated the studies at each stage. Any disagreements regarding study selection were resolved through discussion.

### Data Extraction

Two investigators (HJC and GJY) independently reviewed the full texts of included studies. Data extraction was performed independently by both reviewers, capturing key details, including first author’s name, the age of the participants, time after stroke, the number of participants by different hemiplegic sides and stroke types, publication year, BCI signal acquisition method, feedback device, sample size, intervention details, intervention duration, outcome measures, and follow-up period. For studies reporting outcomes solely as median and IQR, these values were converted to estimated mean and SD using the methods recommended by the Cochrane Handbook [26]: mean ≈ median; SD ≈ IQR / 1.35 [9]. Any discrepancies between reviewers were initially resolved through discussion. Persistent disagreements were resolved through discussion.

### Quality Assessment

According to the PRISMA guidelines [23], the risk of bias assessment was conducted for the included RCTs. Two investigators (HJC and GJY) independently assessed the methodological quality and risk of bias for included studies using the Cochrane Risk of Bias tool (RoB 2.0) [27]. The RoB 2.0 evaluates five domains: (1) bias arising from the randomization process; (2) bias due to deviations from intended interventions; (3) bias due to missing outcome data; (4) bias in outcome measurement; and (5) bias in selection of the reported result. Studies were rated as superior if all domains were judged at low risk, indicating minimal bias concerns. Studies with some domains at low risk but others raising concerns were rated good, reflecting a moderate risk of bias. Studies where no domain achieved low risk were rated poor, indicating substantial bias concerns. Disagreements between assessors were resolved through discussion.

Additionally, the overall quality of evidence for each primary outcome was assessed using the GRADE (Grading of Recommendations Assessment, Development and Evaluation) framework [28]. Two reviewers (HJC and GJY) independently evaluated the evidence quality for the following outcomes. The GRADE approach considers 5 domains, including risk of bias, inconsistency, indirectness, imprecision, and publication bias. Evidence quality was categorized as high, moderate, low, or very low. Any discrepancies in ratings were resolved through discussion.

### Statistical Analyses

Statistical analysis was performed using Stata 18 (StataCorp LLC) software. Data were pooled using mean differences (MDs) with 95% CIs to assess BCI training efficacy. The extent of heterogeneity was quantified using the tau-square (τ²) statistic, which estimates the variance of true effect sizes across studies. The *I*² statistic is reported as a supplementary measure, representing the percentage of total variability in effect estimates that is due to heterogeneity rather than sampling error [29,30]. The Cochran Q test (chi-square test) was used to test the null hypothesis of homogeneity; a significant *P* value (*P*<.05) was taken as evidence of the presence of heterogeneity [31]. Given the anticipated clinical and methodological diversity among the included studies, all meta-analyses were performed using the random-effects model. To ensure more robust and conservative estimates, especially given the varying number of studies included in different analyses, we applied the Hartung-Knapp-Sidik-Jonkman adjustment for calculating the 95% CIs around the pooled MDs [32]. This model provides a more conservative estimate of the effect size and its CI by accounting for both within-study and between-study variability. To quantify the implications of heterogeneity and estimate the range within which the true effect size is likely to fall in future similar scenarios, we calculated 95% prediction intervals (PIs) for the meta-analyses of all outcome measures included in this systematic review so as to ensure the reliability of the estimation of between-study variance [33].

The outcome measures of our meta-analysis included upper limb motor function, muscle tone, and ADL. The primary outcome measure was the FMA-UE, which is a commonly used indicator for clinical assessment of upper limb dysfunction in people who have experienced a stroke [34]. Specifically, the ARAT served as the assessment scale for upper limb function, the MAS was used for muscle tone evaluation, and both the MBI and MAL were used to assess ADL in people who have experienced a stroke.

Subgroup analyses were performed to assess the influence of the following potential factors on upper limb functional outcomes:

1. Feedback modality: BCI-FES, BCI-robot, or BCI-visual feedback;
2. Intervention intensity: stratified by session duration (20 min vs 30 min vs 60 min), weekly frequency (2-3 days vs 4-5 days), total intervention duration (2 weeks vs 4-5 weeks vs 8 weeks), and total number of sessions (10-12 times vs 20-24 times);
3. Follow-up time point: short-term (≤3 months) vs long-term (>3 months).

The thresholds for these subgroup classifications were defined based on the most frequently reported intervention parameters across the included studies and common dosing regimens in prior clinical research [35,36]. This categorization allows for a direct comparison of the different training intensities most commonly encountered in the current evidence base.

Sensitivity analysis was performed to investigate potential sources of heterogeneity. Assessment of small-study effects was performed using funnel plots and the Egger test for outcomes that included a sufficient number of studies (typically n>10). A *P* value <.05 in the Egger test was considered indicative of potential small-study effects [37].

## Results

### Search Results

**Figure 1:**
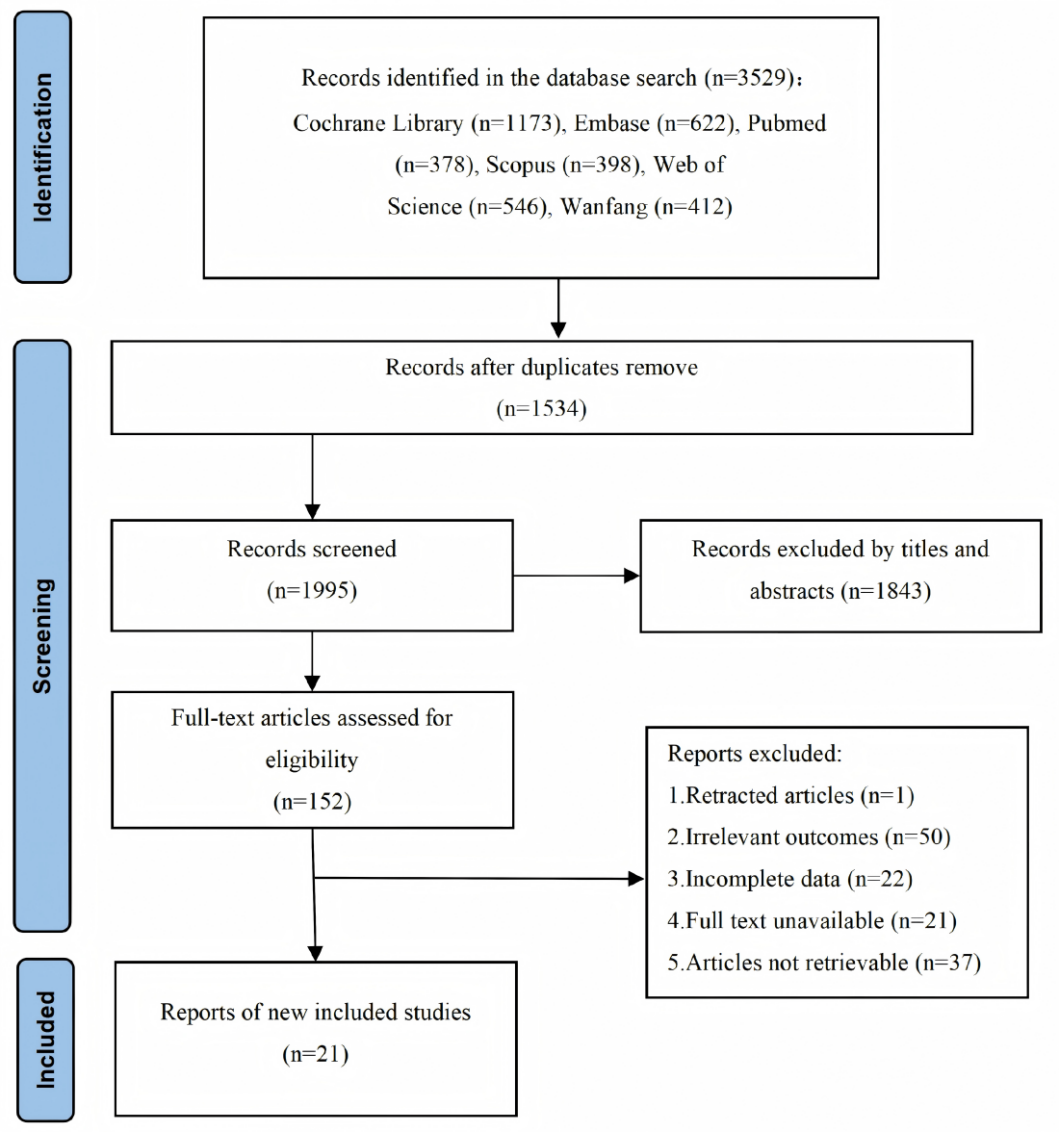
PRISMA (Preferred Reporting Items for Systematic Reviews and Meta-Analyses) flow diagram of the study selection process.

The systematic literature search yielded a total of 3529 records from the 6 databases Cochrane Library (n=1173), Embase (n=622), PubMed (n=378), Scopus (n=398), Web of Science (n=546), and Wanfang (n=412). Following the deduplication process as prespecified in the methods, 1534 duplicate publications were removed. The remaining 1995 unique records underwent initial title and abstract screening. Subsequently, 152 articles were selected for full-text assessment, from which 21 studies met the predefined inclusion criteria and were included in the final meta-analysis. The study selection process is detailed in the PRISMA flow diagram (Figure 1).

### Quality Evaluation

**Figure 2:**
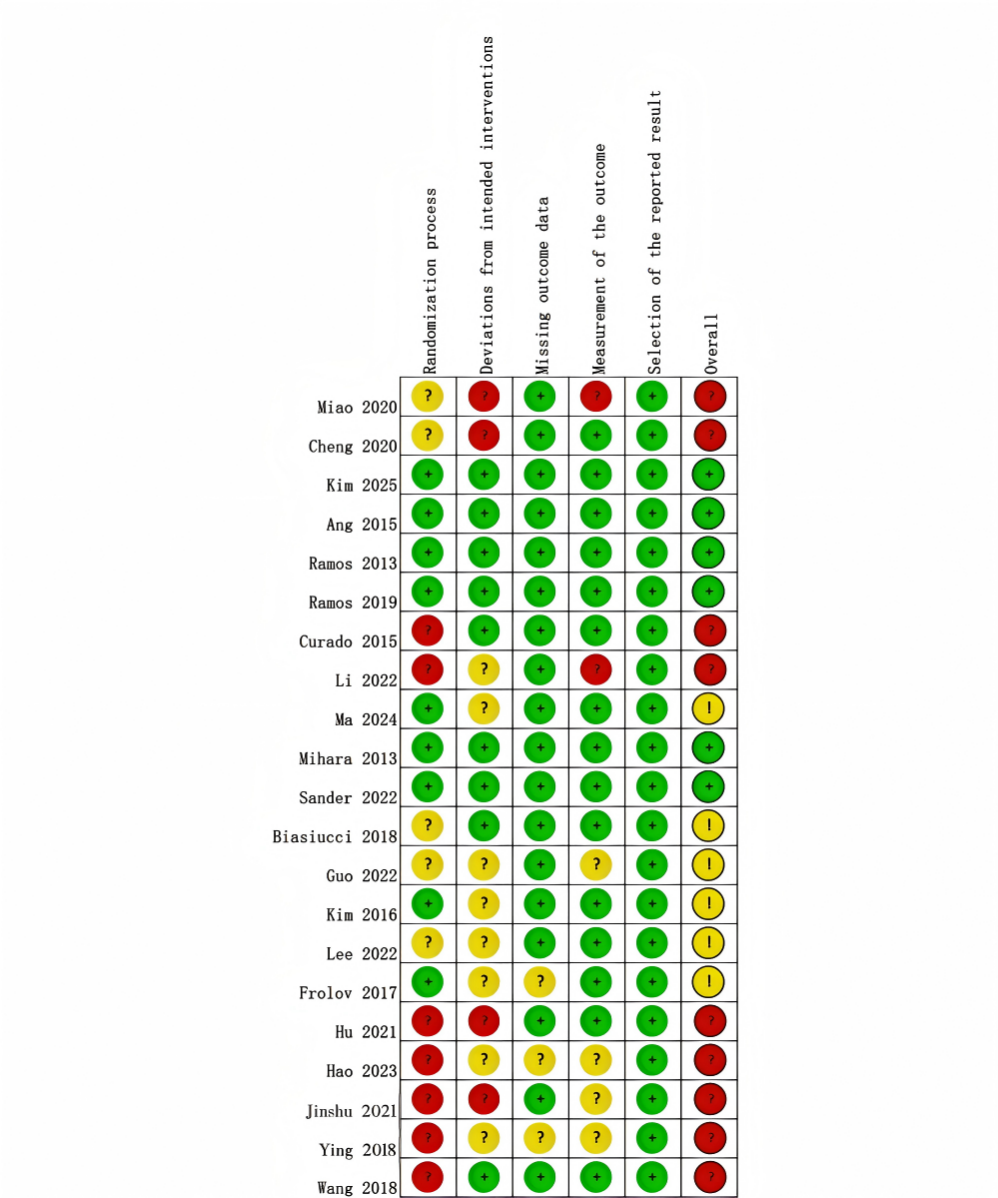
Risk of bias assessment for included randomized controlled trials (RoB 2.0) [11,19,35,38-55].

The methodological quality of the 21 included RCTs was assessed using the revised RoB 2. Based on this assessment, 6 RCTs were classified as “Superior,” 6 as “Good,” and 9 as “Poor.” In several studies, the substantial differences in interventions between the experimental and control groups made blinding of participants and intervention providers not feasible. Consequently, these studies were judged to carry a high risk of bias. The specific evaluation results are shown in Figure 2 [11,19,35,38-55].

The overall quality of evidence for the primary outcomes, as assessed by the GRADE approach, ranged from low to moderate. The summary of findings and the detailed GRADE evidence profile for each outcome are available in Multimedia Appendix 3.

### Characteristics of the Included Literature

A total of 21 studies [11,19,35,38-55], published between 2013 and 2025, were included in this meta-analysis. These studies comprised 337 participants in experimental groups and 313 in control groups. Individual intervention sessions ranged from 20 minutes to 120 minutes, while the total intervention duration varied from 3 days to 10 weeks, encompassing 6-70 sessions in total. Follow-up assessments were performed in 8 studies [11,19,39,42,45-47,55]. Regarding the BCI feedback modality, FES was used in 7 studies [19,38,40,48,49,52,54], exoskeleton devices in 10 studies [11,39,41-44,47,50,53,55], and visual feedback in 4 studies [35,45,46,51]. Specific methods for random sequence generation were described in 15 studies [11,19,35,40-42,44-46,48-50,52-54]. Allocation concealment was implemented in 8 studies [11,19,40-42,45,46,48], and outcome assessors were blinded in 15 studies [11,19,35,39-43,45,46,48-51,55]. The detailed characteristics of the included studies are presented in Multimedia Appendix 4.

### Results of Meta-Analysis

#### Effect of BCI on Overall Motor Function (FMA-UE)

**Figure 3:**
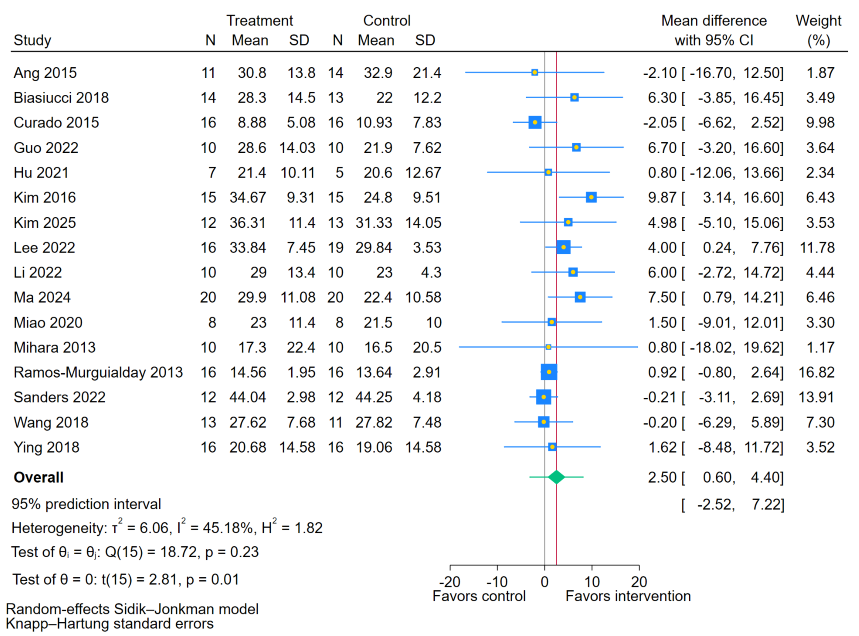
Forest plot for the effect of brain-computer interface (BCI) training on Fugl-Meyer Assessment for upper extremity (FMA-UE) scores [11,19,35,38,40,41,43-49,51,54,55].

As illustrated in Figure 3, sixteen [11,19,35,38,40,41,43-49,51,54,55] studies reported the effects of BCI training on FMA-UE scores in people with chronic stroke. The meta-analysis demonstrated a statistically significant improvement in FMA-UE scores following BCI training (MD 2.50, 95% CI 0.60-4.40; *P*=.01). The test for heterogeneity was not statistically significant (Q=18.72; *P*=.23), and the *I*² was 45.18%. The estimated between-study variance was tau²=6.06. The 95% PI was (–2.52 to 7.22), indicating that the effect of BCI on FMA-UE in a future similar study could range from a clinically irrelevant decline of 2.52 points to a clinically meaningful improvement of 7.22 points.

#### Effect of BCI on Fine Motor Skills (ARAT)

**Figure 4:**
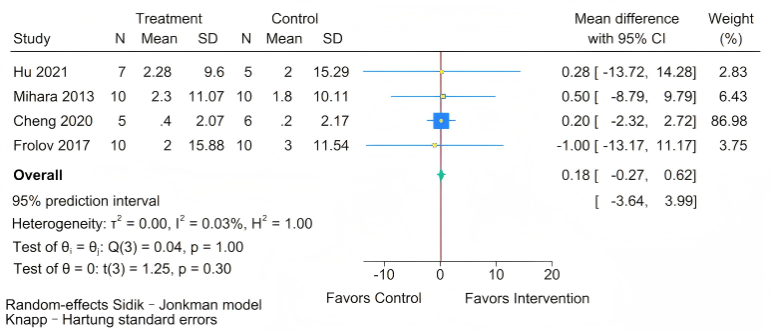
Forest plot for the effect of brain-computer interface (BCI) training on Action Research Arm Test (ARAT) scores [39,45,50,51].

As depicted in Figure 4, four [39,45,50,51] studies evaluated the impact of BCI training on ARAT scores in people with chronic stroke. The meta-analysis revealed no statistically significant difference in ARAT scores between the intervention and control groups (MD=0.18, 95% CI –0.27 to 0.62; *P*=.30). The test for homogeneity was not statistically significant (Q=0.04; *P*=.99). The estimated between-study variance was tau²=0. The *I*² statistic was 0.03%. Furthermore, the 95% PI was (–3.64 to 3.99). This wide interval, which spans both clinically negligible negative and positive effects, underscores the considerable uncertainty regarding the true effect of BCI on fine motor skills and indicates that in future settings, the outcome could range from a slight worsening to a modest improvement.

#### Effect of BCI on Muscle Tone (MAS)

**Figure 5:**
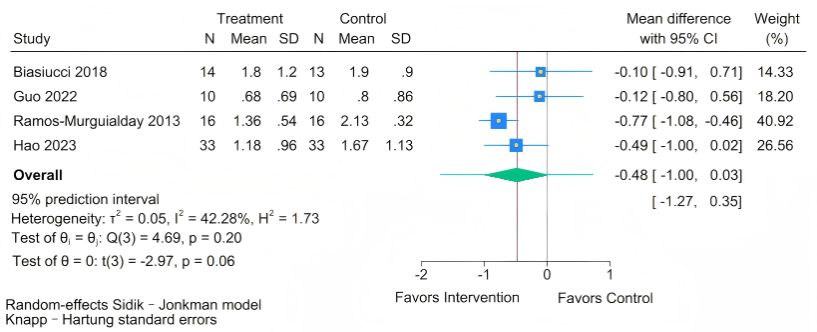
Forest plot for the effect of brain-computer interface (BCI) training on Modified Ashworth Scale (MAS) scores [19,41,47,52].

As presented in Figure 5, four [19,41,47,52] studies assessed the effect of BCI training on upper limb muscle tone in people with chronic stroke. The meta-analysis demonstrated no statistically significant difference in muscle tone outcomes between the BCI and control groups (MD –0.48, 95% CI –1 to 0.03; *P*=.06). The test for homogeneity was not statistically significant (Q=4.69; *P*=.20). The estimated between-study variance was tau²=0.05. The *I*² statistic was 42.28%. However, the 95% PI was (–1.27 to 0.35), offering a more nuanced interpretation. As lower scores on the MAS indicate reduced spasticity, this interval suggests that in future clinical settings, the effect of BCI on muscle tone is predicted to range from a small but potentially meaningful reduction to a negligible change.

### Effects on ADL

#### Effect of BCI on Activities of Daily Living (MBI)

**Figure 6:**
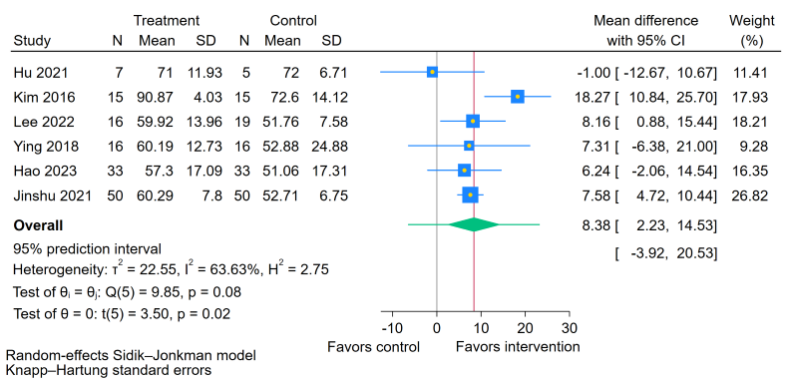
Forest plot for the effect of brain-computer interface training on Modified Barthel Index (MBI) scores [48,49,51-54].

As illustrated in Figure 6, six [48,49,51-54] studies evaluated the effects of BCI training on MBI scores in people with chronic stroke. The meta-analysis demonstrated a statistically significant improvement in MBI scores following BCI intervention (MD 8.38, 95% CI 2.23-14.53; *P*=.02). The test for homogeneity was not statistically significant (Q=9.85; *P*=.08). However, the *I*² statistic was 63.63%, and the estimated between-study variance was tau²=22.55, indicating substantial heterogeneity in the magnitude of the effect across studies. The 95% PI was (–3.92 to 20.53), suggesting substantial uncertainty in the magnitude of the effect across different clinical settings, with effects potentially ranging from negligible to substantially beneficial.

#### Effect of BCI on Self-Reported Arm Use (MAL)

**Figure 7:**
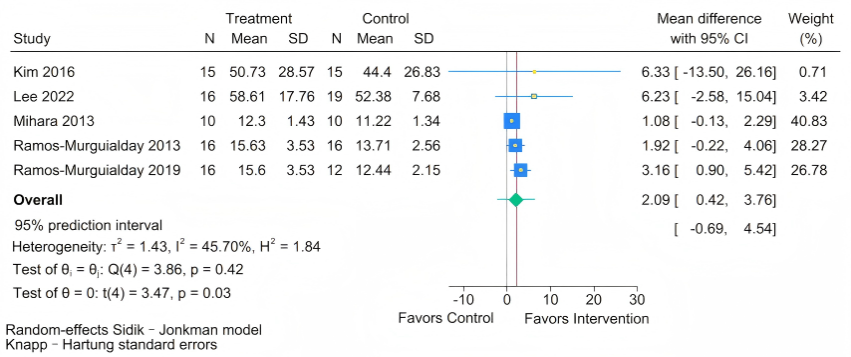
Forest plot for the effect of brain-computer interface (BCI) training on Motor Activity Log (MAL) scores [41,42,45,48,49].

As shown in Figure 7, five [41,42,45,48,49] studies examined the effect of BCI training on MAL scores in people with chronic stroke. The meta-analysis showed a statistically significant improvement in MAL scores following BCI intervention (MD 2.09, 95% CI 0.42-3.76; *P*=.03). The test for homogeneity was not statistically significant (Q=3.86; *P*=.42). The estimated between-study variance was tau²=1.43. The *I*² statistic was 45.7%. The 95% PI was (–0.69 to 4.54). This indicates that while the average effect is positive, the true effect in a new setting could range from a negligible or slightly negative impact to a substantial improvement in patient-perceived arm use during daily activities. The fact that the majority of the interval lies above zero strengthens the evidence for a likely beneficial effect, albeit of variable magnitude.

### Subgroup Analysis: Feedback

**Figure 8:**
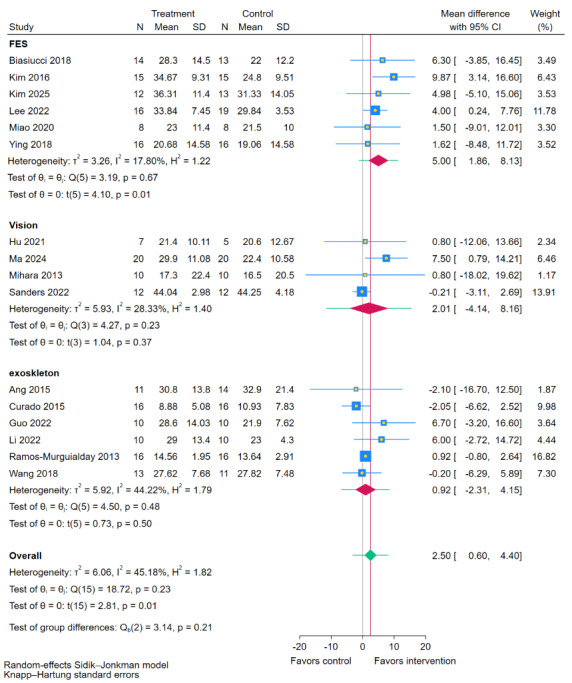
Subgroup analysis of Fugl-Meyer Assessment for upper extremity (FMA-UE) scores by brain-computer interface (BCI) feedback modality [11,19,35,38,40,41,43-49,51,54,55].

In the subgroup analysis stratified by BCI feedback modality (Figure 8 [11,19,35,38,40,41,43-49,51,54,55]), the test for subgroup differences indicated no statistically significant difference between the modalities (*P*=.21). However, within-subgroup analyses revealed that only the BCI-FES paradigm showed a significant improvement in FMA-UE scores compared to routine rehabilitation therapy (RRT: MD 5, 95% CI 1.86-8.13; *P*=.01). In contrast, neither the BCI-Exoskeleton (MD 0.92, 95% CI –2.31 to 4.15; *P*=.50) nor the BCI-Visual (Beijing Intelligent Brain Science and Technology Co, Ltd) feedback (MD 2.01, 95% CI –4.14 to 8.16; *P*=.37) subgroups demonstrated significant effects. Although the differences between feedback modalities were not statistically significant, BCI-FES may be associated with greater motor recovery relative to RRT than the other modalities.

### Intervention Intensity: Session Duration

**Figure 9:**
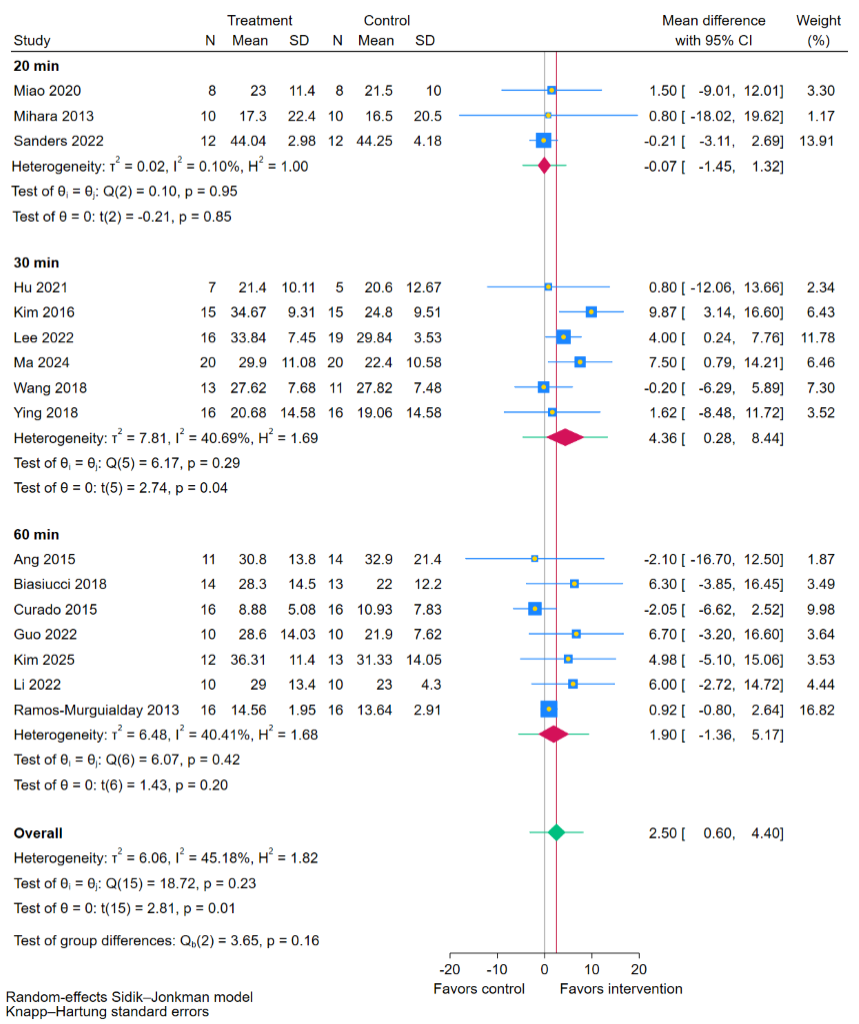
Subgroup analyses of Fugl-Meyer Assessment for upper extremity (FMA-UE) scores by session duration [11,19,35,38,40,41,43-49,51,54,55].

Subgroup analysis based on single-session intervention duration (Figure 9 [11,19,35,38,40,41,43,44,47-49,51,54,55]) demonstrated no statistically significant difference between session duration subgroups (*P*=.16). Within the subgroups, a regimen of 30-minute sessions elicited a significant improvement in FMA-UE scores compared to RRT (MD 4.36, 95% CI 0.28-8.44; *P*=.04), whereas sessions lasting 20 minutes (MD –0.07, 95% CI –1.45 to 1.32; *P*=.85) or 60 minutes (MD 1.90, 95% CI –1.36 to 5.17; *P*=.20) did not. These results suggest that while the difference between session durations was not statistically significant, a 30-minute session may be associated with optimal outcomes.

### Training Sessions per Week

**Figure 10:**
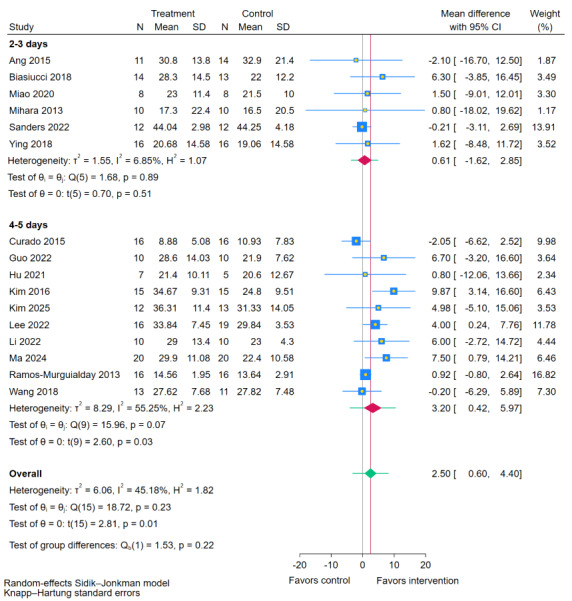
Subgroup analyses of Fugl-Meyer Assessment for upper extremity (FMA-UE) scores by training sessions per week [11,19,35,38,40,41,43-49,51,54,55].

Subgroup analysis based on weekly intervention frequency (Figure 10 [11,19,35,38,40,41,43,44,47-49,51,54,55]) found no statistically significant difference between the frequency subgroups (*P*=.22). The higher-frequency regimen (4-5 sessions per week) was associated with a significant improvement in FMA-UE scores compared to RRT (MD 3.20, 95% CI 0.42-5.97; *P*=.03). The lower-frequency regimen (2-3 sessions per week) did not show a significant effect (MD 0.61, 95% CI –1.62 to 2.85; *P*=.51). This indicates that while the difference between weekly frequencies was not statistically significant, a higher frequency of 4-5 sessions per week may be linked to better motor recovery.

#### Duration of Intervention

**Figure 11:**
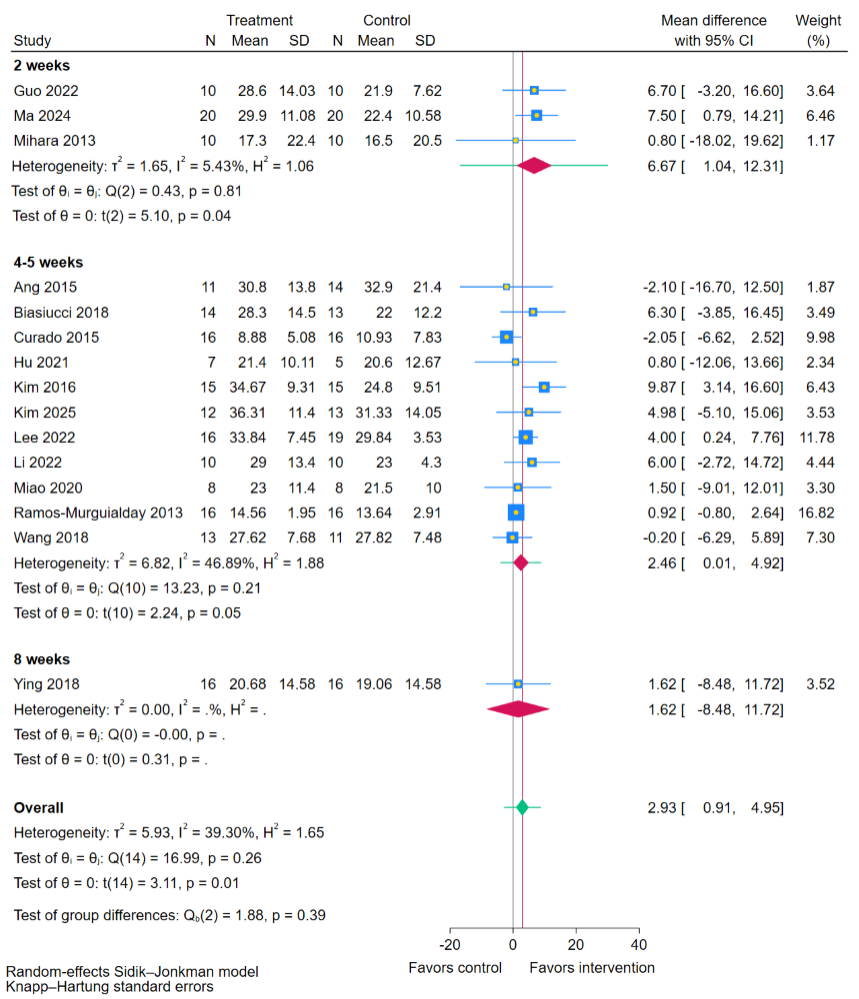
Subgroup analyses of Fugl-Meyer Assessment for upper extremity (FMA-UE) scores by duration of intervention [11,19,35,38,40,41,43-45,47-49,51,54,55].

Subgroup analysis based on the total intervention duration (Figure 11 [11,19,35,38,40,41,43-45,47-49,51,54,55]) showed no statistically significant difference between the duration subgroups (*P*=.39). Within the subgroups, a shorter-duration regimen of 2 weeks elicited a significant improvement in FMA-UE scores compared to RRT (MD 6.67, 95% CI 1.04-12.31; *P*=.04). Interventions lasting 4-5 weeks (MD 2.46, 95% CI 0.01-4.92; *P*=.05) or 8 weeks (MD 1.62, 95% CI –8.48 to 11.72) did not show significant effects. This suggests that although the difference between intervention durations was not statistically significant, a shorter, more concentrated 2-week period may be associated with superior efficacy.

#### Total Number of Sessions

**Figure 12:**
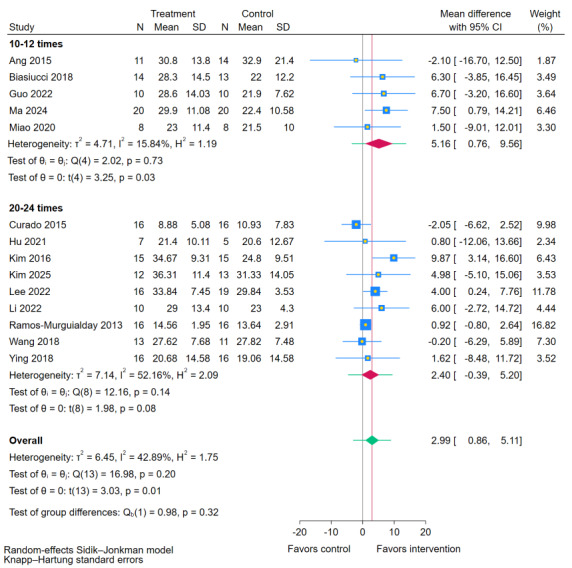
Subgroup analyses of Fugl-Meyer Assessment for upper extremity (FMA-UE) scores by total number of sessions [11,19,35,38,40,41,43,44,47-49,51,54,55].

Subgroup analysis stratified by the total number of intervention sessions (Figure 12 [11,19,35,38,40,41,43,44,47-49,51,54,55]) indicated no statistically significant difference between the session count subgroups (*P*=.32). A lower total session count (10-12 sessions) was associated with a significant improvement in FMA-UE scores compared to RRT (MD 5.16, 95% CI 0.76-9.56; *P*=.03). A higher session count (20-24 sessions) did not demonstrate a significant effect (MD=2.40, 95% CI –0.39 to 5.20; *P*=.08). These findings imply that while the difference between total session numbers was not statistically significant, a protocol comprising 10-12 sessions may be linked to more favorable outcomes.

#### Follow-Up

**Figure 13:**
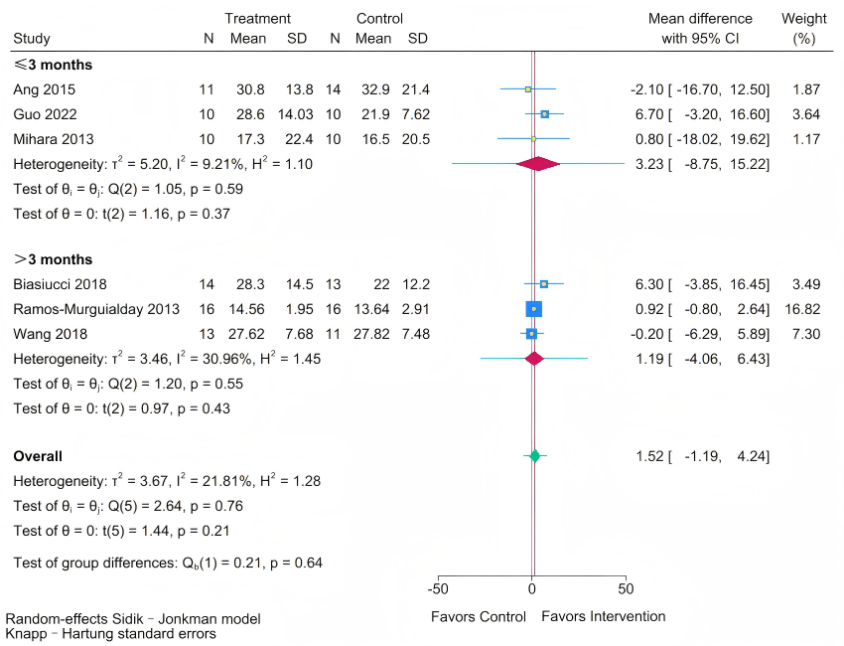
Subgroup analysis of Fugl-Meyer Assessment for upper extremity (FMA-UE) scores by follow-up period [11,19,41,45,47,55].

Analysis of follow-up outcomes (Figure 13 [11,19,41,45,47,55]) revealed no significant differences in long-term FMA-UE improvement between BCI-based training and RRT at any follow-up interval, whether assessed at short-to-medium term (≤3 months; MD 3.23, 95% CI –8.75 to 15.22; *P*=.37) or long-term (>3 months; MD 1.19, 95% CI –4.06 to 6.43; *P*=.43).

### Sensitivity Analysis

A leave-one-out sensitivity analysis was performed to assess the robustness of the pooled MBI results and to investigate the influence of individual studies on the substantial observed heterogeneity (initial *I*²=63.63%; τ²=22.55).

**Figure 14:**
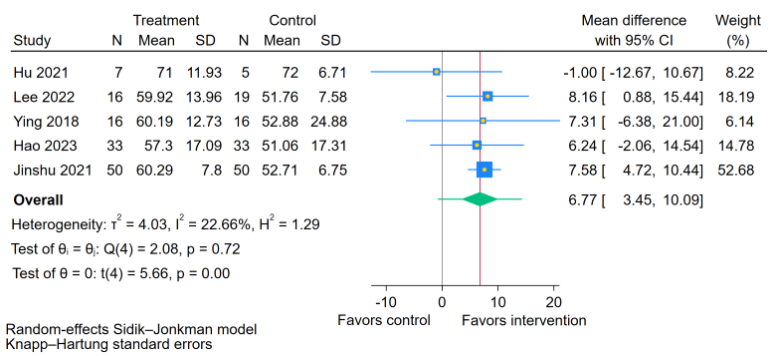
Sensitivity analysis for the effect of brain-computer interface (BCI) training on Modified Barthel Index (MBI) scores [49,51-54].

As shown in Figure 14 [49,51-54], the sequential exclusion of each study revealed that the findings were robust overall. However, the exclusion of a single study—Kim et al [48]—led to a marked reduction in heterogeneity, with the *I*² statistic decreasing from 63.63% to 22.66% and the tau² value dropping from 22.55 to 4.03. Crucially, the pooled estimate remained statistically significant and the CI narrowed, indicating increased precision (MD 6.77, 95% CI 3.45-10.09; *P*<.001), compared to the original analysis (MD 8.38, 95% CI 2.23-14.53; *P*=.02). This suggests that while the study by Kim et al [48] was a major contributor to the statistical heterogeneity, the conclusion that BCI training improves ADL is robust.

### Small-Study Effects Analysis

**Figure 15:**
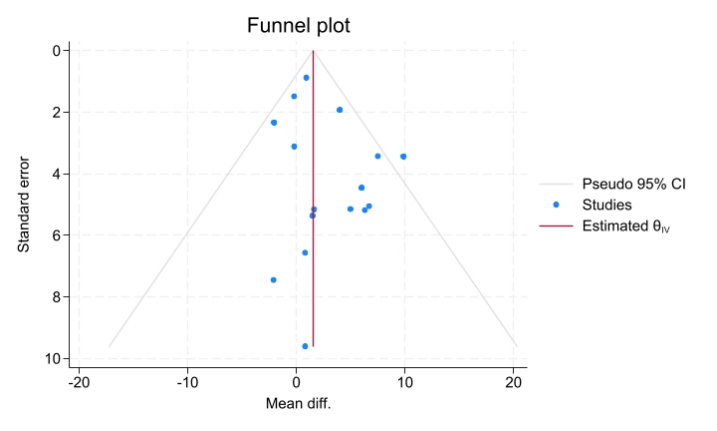
Funnel plot assessing small-study effects for the Fugl-Meyer Assessment for upper extremity (FMA-UE) outcome.

Within this analysis, only the FMA-UE outcome pooled a sufficient number of studies (n>10) for the assessment of small-study effects. Accordingly, a funnel plot was constructed for the FMA-UE outcome (Figure 15). Visual inspection revealed no substantial asymmetry. Furthermore, the Egger regression test yielded a nonsignificant result (*P*=.20), suggesting no strong evidence for small-study effects for this outcome.

## Discussion

### Overview

This meta-analysis differs from the general efficacy evaluation of BCI in the treatment of chronic stroke. By conducting an in-depth analysis of how treatment parameters influence prognosis, it has deepened the understanding of this therapeutic approach. While the overall benefits of BCI for upper limb motor function and ADLs are confirmed, our meta-analysis attempts to offer greater clinical utility by identifying the specific feedback modality (BCI-FES) and a distinct, time-efficient training schedule associated with optimal recovery. These findings contribute to optimizing BCI intervention protocols and promoting their clinical implementation, advancing the translation of this technology between experimental applications and real-world clinical practice.

### Summary of Main Findings

The meta-analysis findings demonstrate that BCI training yields benefits in improving overall upper limb motor function and ADLs in patients with chronic stroke. Specifically, BCI interventions led to significant improvements in the primary outcome of motor impairment (FMA-UE) and in patient-reported and performance-based measures of daily function (MBI and MAL). In contrast, BCI training did not yield significantly superior effects on fine motor function (ARAT) or muscle tone (MAS) compared to control interventions.

### Clinical Significance and Heterogeneity of Effects

Building on previous meta-analyses [9,56], our findings reveal a statistically significant incremental improvement in FMA-UE scores over conventional therapy. While this gain alone falls below the minimal clinically important difference threshold of approximately 5 points [57], it represents a meaningful augmentation to the foundational improvements from standard care. This is particularly noteworthy given the significant enhancements in MBI and MAL scores. This convergence suggests that BCI’s closed-loop methodology, integrating central neural signals with peripheral feedback, may provide a synergistic effect that more effectively translates into functional gains in daily activities.

Furthermore, while the 95% CIs for the FMA-UE, MBI, and MAL confirm a positive average benefit of BCI over control interventions, the prediction intervals (FMA-UE: –2.52 to 7.22; MBI: –3.92 to 20.53; MAL: –0.69 to 4.54) reveal a more complex scenario. These intervals suggest that in future clinical settings, the effect of BCI compared to RRT could range from negligible or even slightly adverse to substantial improvements meeting the minimal clinically important difference. This indicates that current BCI training may be insufficient to yield reliable therapeutic effects for all patients in the chronic phase. The considerable heterogeneity observed suggests that the efficacy of BCI training is not uniform and may be influenced by individual patient conditions or differences in BCI treatment protocols. In line with this, a study by Guo et al [47] also emphasizes that future research should prioritize identifying patient subgroups most likely to benefit from this therapy, specifically, those with effect sizes at the upper end of the prediction interval, such as patients retaining partial integrity of the corticospinal tract.

### Lack of Effect on Fine Motor Control and Spasticity

The absence of significant improvement in fine motor function, as measured by the ARAT, and in muscle tone, assessed by the MAS, warrants further mechanistic consideration. The ARAT primarily evaluates distal upper limb functions, such as grasp, grip, and pinch. The nonsignificant findings may stem from a fundamental limitation of current BCI paradigms, which often decode neural correlates of gross motor imagery (eg, whole-arm reaching or hand opening/closing) rather than the finely graded, individuated movements required for dexterous tasks. The feedback provided, particularly via exoskeleton or visual modalities, may lack the specificity necessary to engage and reinforce the delicate cortical representations of the hand.

The PI for the ARAT (–3.64-3.99) provides a deeper perspective on this null result. This interval is not only symmetrically distributed around the null effect but also entirely excludes the possibility of any large, clinically meaningful positive effects. However, given that only 6 of the included studies were rated as having a low risk of bias and the GRADE assessment indicated moderate-quality evidence for the ARAT outcome, this conclusion must be interpreted with caution.

Consequently, clinicians should be cautious about prioritizing the improvement of fine motor function as a primary goal when applying current mainstream BCI paradigms. Future optimization of BCI systems should focus on enhancing the decoding resolution of finer motor intentions, potentially by using high-density EEG or hybrid BCI approaches, and by integrating hand-specific training adjuncts such as virtual reality environments with object manipulation or wearable devices providing tactile or proprioceptive feedback to the distal limb.

Similarly, the lack of a significant effect of BCI on muscle tone suggests that its primary mechanism of action likely involves facilitating active motor control and cortical reorganization, rather than directly modulating the spinal reflex pathways underlying spasticity. In chronic stroke, hypertonia is often well-established, necessitating targeted interventions. While BCI can promote Hebbian plasticity through the associative pairing of motor intention and movement execution, this effect may be insufficient to reverse impaired supraspinal inhibitory control over the spinal motor pool.

Nevertheless, the 95% PI for the MAS (–1.27 to 0.35) provides valuable clinical insight. As lower scores indicate reduced spasticity, this interval—spanning from “no change” to “improvement”—suggests that BCI therapy is unlikely to exacerbate spasticity in future applications. Moreover, its lower bound of –1.27 indicates that under specific conditions, such as when combined with certain forms of FES, BCI may yield meaningful reductions in muscle tone. This potentially “non-harmful” profile, particularly when considered alongside the moderate quality of the existing evidence, represents an important factor for clinical decision-making and offers a preliminary rationale for exploring BCI as a component of comprehensive spasticity management protocols.

### Superiority of BCI-FES and the Role of Feedback Modality

Subgroup analyses revealed that only the BCI-FES paradigm demonstrated significantly greater improvement in FMA-UE compared to control. This superiority can be explained through the lens of neuroplasticity and sensorimotor integration. The BCI-FES paradigm creates a closed-loop system that tightly couples motor intention with peripheral afferent feedback. According to Hebbian learning principles [58], which posit that “neurons that fire together, wire together,” the synchronous activation of the motor cortex (during attempted movement imagery) and the somatosensory cortex (via FES-induced limb movement and proprioceptive input) strengthens the synaptic connections within the sensorimotor network, aligning with findings from the meta-analysis by Li et al [9].

The lack of significant benefit with BCI-Exoskeleton may relate to factors such as device complexity, comfort limitations, and suboptimal anatomical fit. Furthermore, excessive robotic assistance could potentially reduce patient engagement and diminish the crucial training effect driven by active neural effort [59]. The negative results with BCI-Vision indicate that visual feedback alone, in the absence of concomitant somatosensory input and actual limb movement, may have limited efficacy in driving neural reorganization and functional recovery, particularly in patients with chronic deficits or more severe functional impairments.

### Sustainability of Benefits and the Need for Maintenance

Subgroup analysis based on follow-up duration revealed no significant sustained advantage of BCI over control after the active treatment phase ceased. This implies that the functional gains may lack long-term stability without ongoing application.

In chronic patients, BCI may effectively induce neuroplasticity during the intensive training period, potentially by reinforcing specific neural pathways or facilitating compensatory mechanisms. However, these newly formed connections or patterns might lack sufficient stability or robustness. Following intervention cessation, without ongoing functional application or specific maintenance training, these acquired neural adaptations may gradually regress or weaken. Conversely, standard RRT protocols often inherently incorporate recommendations for continued activity.

These findings highlight the necessity for systematic maintenance strategies—such as telerehabilitation, behavioral incentive programs, or continued use of assistive technologies—to be integrated postintervention. Addressing this challenge of sustained efficacy represents a crucial future research direction.

### Toward an Optimal and Efficient Intervention Protocol

Although tests for subgroup differences were not statistically significant, the within-subgroup comparisons revealed a consistent and clinically meaningful pattern favoring specific parameters. The most robust finding is the efficacy of a short-term, high-density protocol.

This paradigm—comprising sessions of approximately 30 minutes each, delivered 4-5 times per week over a total of 10-12 sessions (approximately 2 weeks)—yielded optimal FMA-UE outcomes compared to control interventions. While broader intensity subgroup comparisons (eg, session duration or total weeks) were not statistically significant, this specific, condensed protocol was the only intensity paradigm that consistently demonstrated a significant within-subgroup effect. This finding underscores the potential primacy of training density—the concentration of practice within a shorter timeframe—over the total intervention duration [50]. Furthermore, a higher frequency of 4-5 sessions per week proved superior to regimens of 2-3 sessions per week, indicating that more frequent exposure facilitates sharper motor patterns and stronger memory traces [60].

Notably, completing 10-12 sessions within 2 weeks was more effective than protocols delivering 20-24 sessions over 4-5 weeks. This observation strongly supports the established concept that maximizing neuroplasticity in people with chronic stroke often requires intensive, repetitive, and focused training to overcome neural inhibition and promote synaptic strengthening [61].

Therefore, based on the current evidence, a protocol of 30-minute sessions, administered 4-5 times per week over 2 weeks (totaling 10-12 sessions), emerges as a promising and efficient model for BCI intervention. It is crucial to emphasize that this proposal is not intended as a definitive guideline but rather highlights a potentially optimal treatment paradigm derived from the existing, albeit limited, data. This model warrants prioritization and validation in future rigorous research.

### Research Prospects

Although subgroup analyses favor short-term, high-frequency protocols, this intensity may be insufficient to induce lasting neuroplastic reorganization. This observation aligns with the dissipation of functional gains at follow-up. Future high-quality RCTs are required to (1) delineate the dose-response relationship of BCI training, (2) analyze the synergistic effects of BCI combined with complementary therapies to optimize rehabilitation protocols, and (3) evaluate efficacy differentials based on lesion characteristics and upper-limb impairment severity in patients with chronic disease, thereby identifying responsive subpopulations. These investigations aim to inform evidence-based rehabilitation strategies and research priorities for stroke recovery.

### Limitations

Several limitations warrant consideration in this meta-analysis. First, while the included 21 studies underwent rigorous quality assessment using the Cochrane RoB 2 tool, only 6 were rated as having “Low” risk of bias. This relatively low proportion of high-quality studies may limit the robustness of our findings. Second, insufficient studies (fewer than 5) were included in some subgroup analyses, potentially compromising the reliability of conclusions drawn for those specific comparisons. Third, the subgroup analyses, particularly for intervention intensity, were likely underpowered to detect statistically significant differences between parameters due to the limited number of studies in each category. Therefore, the identified optimal protocol should be viewed as the most evidence-based recommendation from the current data, rather than a definitively proven superior approach. Future research should prioritize incorporating a greater number of high-quality RCTs. Furthermore, greater emphasis is needed on exploring the impact of BCI training intervention intensity and focusing on outcomes such as improvements in muscle tone and standardized assessments like the ARAT. Investigating these areas represents promising avenues for advancing stroke rehabilitation.

### Conclusion

Low- to moderate-certainty evidence suggests that BCI training, particularly the BCI-FES paradigm, can improve upper limb motor function and ADL in people with chronic stroke on average. However, wide prediction intervals indicate the effect may vary substantially across settings, ranging from negligible to beneficial. Subgroup analyses suggested a potential optimal protocol of 30-minute sessions, 4-5 times per week for 2 weeks, but these findings are limited by the small number of studies in each subgroup and the high risk of bias in several included trials. Therefore, this proposed protocol should be viewed as preliminary and requires validation in future, high-quality RCTs. Future research should also focus on identifying patient subgroups most likely to benefit and on strategies to sustain long-term gains.

## Appendix

Multimedia Appendix 1 PRISMA checklist.

Multimedia Appendix 2 Search strategy.

Multimedia Appendix 3 GRADE assessment.

Multimedia Appendix 4 The characteristics of included literatures.

## Abbreviations

ADL: activities of daily living
ARAT: Action Research Arm Test
BCI: brain-computer interface
EEG: electroencephalography
FES: functional electrical stimulation
FMA-UE: Fugl-Meyer Assessment for upper extremity
fNIRS: functional Near-Infrared Spectroscopy
GRADE: Grading of Recommendations Assessment, Development and Evaluation
MAL: Motor Activity Log
MAS: Modified Ashworth Scale
MBI: Modified Barthel Index
MD: mean difference
MeSH: Medical Subject Headings
PI: prediction interval
PRESS: Peer Review of Electronic Search Strategies
PRISMA: Preferred Reporting Items for Systematic Reviews and Meta-Analyses
PRISMA-S: Preferred Reporting Items for Systematic Reviews and Meta-Analyses – Search Extension
PROSPERO: International Prospective Register of Systematic Reviews
RCT: randomized controlled trial
ROB: Risk of Bias tool
RRT: routine rehabilitation therapy

## Data Availability

The datasets generated or analyzed during this systematic review are available from the corresponding author on reasonable request.
