# Supplementary material for: Efficacy of Brain-Computer Interface Therapy for Upper Limb Rehabilitation in Chronic Stroke: Systematic Review and Meta-Analysis of Randomized Controlled Trials
Source: J Med Internet Res. 2026 Jan 28;28:e79132. doi: 10.2196/79132 (PMC12895162; doi:10.2196/79132)
Supplement: Multimedia Appendix 2 [file jmir_v28i1e79132_app2.docx]

**Multimedia Appendix 1. Full Electronic Search Strategies.**

**Database: PubMed (via NLM)**

**Date Searched: October 16, 2025**

**Records Retrieved: 378**

| **#** | **Search terms** | **Results** |
| --- | --- | --- |
| 1 | ("Stroke"[Mesh] OR "Stroke Rehabilitation"[Mesh] OR "Cerebrovascular Disorders"[Mesh]) OR (stroke[tiab] OR post-stroke[tiab] OR cerebrovascular accident[tiab] OR CVA[tiab] OR hemipleg*[tiab]) | 652,101 |
| 2 | ( "Brain-Computer Interfaces"[Mesh] OR "BCI"[tiab] OR "brain-computer interface"[tiab] OR "brain-machine interface"[tiab] OR "neurofeedback"[tiab] OR "motor imagery"[tiab] OR "EEG-based"[tiab] ) | 19,601 |
| 3 | ("Randomized Controlled Trial"[pt] OR "Controlled Clinical Trial"[pt] OR "randomized"[tiab] OR "randomly"[tiab] OR "placebo"[tiab] OR "trial"[tiab]) | 1,908,783 |
| 4 | #1 AND #2 AND #3 | 378 |

**Database: Scopus (via Elsevier)**

**Date Searched: October 16, 2025**

**Records Retrieved: 398**

| **#** | **Search terms** | **Results** |
| --- | --- | --- |
| 1 | (TITLE-ABS-KEY ("stroke" OR "post-stroke" OR "cerebrovascular accident" OR "cva" OR "hemipleg*") OR INDEXTERMS ("stroke" OR "cerebrovascular accident" OR "hemiplegia")) | 689,310 |
| 2 | (TITLE-ABS-KEY ("bci" OR "brain-computer interface" OR "brain-machine interface" OR "neurofeedback" OR "motor imagery" OR "eeg-based") OR INDEXTERMS ("brain-computer interface")) | 49,683 |
| 3 | TITLE-ABS-KEY ("randomized" OR "randomly" OR "placebo" OR "rct" OR "controlled trial") | 2,304,830 |
| 4 | ((TITLE-ABS-KEY ("stroke" OR "post-stroke" OR "cerebrovascular accident" OR "cva" OR "hemipleg*") OR INDEXTERMS ("stroke" OR "cerebrovascular accident" OR "hemiplegia"))) AND ((TITLE-ABS-KEY ("bci" OR "brain-computer interface" OR "brain-machine interface" OR "neurofeedback" OR "motor imagery" OR "eeg-based") OR INDEXTERMS ("brain-computer interface"))) AND (TITLE-ABS-KEY ("randomized" OR "randomly" OR "placebo" OR "rct" OR "controlled trial")) | 398 |

**Database: Web of Science (via Clarivate Analytics)**

**Date Searched: October 16, 2025**

**Records Retrieved: 546**

| **#** | **Search terms** | **Results** |
| --- | --- | --- |
| 1 | TS=("stroke" OR "post-stroke" OR "cerebrovascular accident" OR "CVA" OR "hemipleg*") | 1,023,883 |
| 2 | TS=("brain-computer interface" OR "BCI" OR "brain-machine interface" OR "neurofeedback" OR "motor imagery" OR "EEG-based") | 48,175 |
| 3 | TS=("randomized" OR "randomly" OR "RCT" OR "controlled trial" OR "placebo") | 2,578,038 |
| 4 | #1 AND #2 AND #3 | 546 |

**Database: Embase (via Ovid)**

**Date Searched: October 16, 2025**

**Records Retrieved: 622**

| **#** | **Search terms** | **Results** |
| --- | --- | --- |
| 1 | 'cerebrovascular accident'/exp OR 'stroke patient'/exp OR 'hemiplegia'/exp OR stroke:ti,ab,kw OR poststroke:ti,ab,kw OR 'cerebrovascular accident':ti,ab,kw OR cva:ti,ab,kw OR hemipleg*:ti,ab,kw | 765,713 |
| 2 | 'brain computer interface'/exp OR bci:ti,ab,kw OR 'brain-computer interface':ti,ab,kw OR 'brain-machine interface':ti,ab,kw OR neurofeedback:ti,ab,kw OR 'motor imagery':ti,ab,kw OR 'eeg-based':ti,ab,kw | 28,221 |
| 3 | 'randomized controlled trial'/de OR 'controlled clinical trial'/de OR randomized:ti,ab,kw OR randomly:ti,ab,kw OR placebo:ti,ab,kw OR trial:ti,ab,kw | 3,059,753 |
| 4 | #1 AND #2 AND #3 | 622 |

**Database: Cochrane Central Register of Controlled Trials (via Wiley)**

**Date Searched: October 16, 2025**

**Records Retrieved: 1173**

| **#** | **Search terms** | **Results** |
| --- | --- | --- |
| 1 | (MH:"Stroke" OR MH:"Cerebrovascular Disorders" OR MH:"Hemiplegia") OR (stroke OR post-stroke OR "cerebrovascular accident" OR CVA OR hemipleg*):ti,ab,kw | 83,006 |
| 2 | (MH:"Brain-Computer Interfaces") OR ("BCI" OR "brain-computer interface" OR "brain-machine interface" OR neurofeedback OR "motor imagery" OR "EEG-based"):ti,ab,kw | 26,052 |
| 3 | (randomized OR randomly OR placebo OR "controlled trial"):ti,ab,kw | 1,495,180 |
| 4 | #1 AND #2 AND #3 | 1,173 |

**Database: Wanfang Data (Wanfang)**

**Date Searched: October 16, 2025**

**Records Retrieved: 412**

| **#** | **Search terms** | **Results** |
| --- | --- | --- |
| 1 | ((主题：("脑机接口" OR "脑机交互" OR "BCI" OR "Brain-Computer Interface" OR "神经反馈")) AND (主题：("中风" OR "脑卒中" OR "卒中" OR "脑血管意外" OR "CVA" OR "Stroke")) AND (主题：("上肢" OR "手臂" OR "手" OR "偏瘫" OR "运动功能" OR "功能恢复" OR "康复"))) | 412 |
