# Supplementary material for: Efficacy of Brain-Computer Interface Therapy for Upper Limb Rehabilitation in Chronic Stroke: Systematic Review and Meta-Analysis of Randomized Controlled Trials
Source: J Med Internet Res. 2026 Jan 28;28:e79132. doi: 10.2196/79132 (PMC12895162; doi:10.2196/79132)
Supplement: Multimedia Appendix 4 [file jmir_v28i1e79132_app4.docx]

**Participants Demographic of Included Studies**

| **study** | **Mean Age in EG(years)** | **Mean Age in CG(years)** | **Time after Stroke**  **(months)** | **Lesion Side(right/left)** | **Stroke type**  **(infarction/hemorrhage) in EG** | **Stroke type**  **(infarction/hemorrhage) in CG** |
| --- | --- | --- | --- | --- | --- | --- |
| Miao et al [38] | Mean:48.75 | Mean:50.25 | EG:18.3±10.9  CG:11.1±5.0 | 9/7 | 4/4 | 5/3 |
| Cheng et al [39] | 62.4±4.7 | 61.4± 4.5 | EG:15.9±10  CG:29.7±8.5 | - | 3/2 | 6/0 |
| Kim et al [40] | 49.0±16.9 | 46.0±12.8 | EG:18.2±16.2  CG:22.2±23.4 | 16/9 | 5/7 | 4/9 |
| Ang et al [11] | 48.5±13.5 | 53.6±9.5 | EG:9.9  CG:9.9 | 11/15 | 5/6 | 4/10 |
| Ramos-Murguialday et al [41] | 61.8±10.1 | 60.6±10.7 | - | - | - | - |
| Ramos-Murguialday [42] | 49.3±12.5 | 50.3±12.2 | - | - | - | - |
| Curado et al [43] | Mean:47 | Mean:50 | EG:66  CG:67.5 | - | - | - |
| Li et al [44] | 60.2±9.3 | 53.5±8.3 | EG:12.5±7.1CG:10.9±7.9 | 9/11 | 7/3 | 3/7 |
| Ma et al [35] | - | - | - | - | - | - |
| Mihara et al [45] | Mean:55.0 | Mean:59.1 | EG:4.5  CG:4.5 | 9/11 | 5/5 | 7/3 |
| Sanders et al [46] | Mean:63.75 | Mean:59.25 | EG:79.33  CG:70.25 | 8/16 | - | - |
| Biasiucci et al [19] | Mean:56.36 | Mean:59.00 | EG:39.79  CG:33.46 | 14/13 | 12/2 | 9/4 |
| Guo et al [47] | \| 60.2±9.3 \| \| --- \| | 53.5±8.3 | EG:12.5±7.1  CG:10.9±7.9 | 9/11 | 7/3 | 3/7 |
| Kim et al [48] | 59.07±8.07 | 59.93±9.79 | EG:8.27±1.98  CG:7.80±1.78 | 14/16 | 8/7 | 6/9 |
| Lee et al [49] | 55.15±11.57 | 58.3± 9.19 | EG:7.46±1.61  CG:8.3±1.97 | - | 8/5 | 7/6 |
| Frolov et al [50] | Mean:58.0 | Mean:58.0 | EG:8  CG:8 | - | - | - |
| Hu et al [51] | 44.9±7.5 | 60.4±16.8 | EG:7.9±6.5; CG:7.3±4.5 | 3/9 | 3/4 | 3/2 |
| Hao et al [52] | 58.45±7.55 | 58.06 ±7.82 | EG:12.36±2.37  CG:10.61±2.75 | - | 19/14 | 18/15 |
| Jinshu et al [53] | 60.35±6.82 | 58.71±7.01 | EG:28±6.9  CG:10±7.2 | - | 35/15 | 33/17 |
| Ying et al [54] | 72.43±8.56 | 76.81±9.57 | - | 12/20 | 13/3 | 14/2 |
| Wang et al [55] | 54±9 | 54±9 | EG:48±36  CG:48±36 | 15/9 | 9/4 | 5/6 |

|  |  |  |  |  |  | | |  |  |
| --- | --- | --- | --- | --- | --- | --- | --- | --- | --- |
| **Baseline Characteristics of Included Studies** | | | | | | | | | |
| **Study** | **Study design** | **country** | **Signal Acquisition** | **Feedback System** | **Intervention Method and Sample size** | | | **Outcome** | **Follow-up** |
|  |  |  |  |  | **EG** | **CG** | **Intervention period** |  |  |
| Miao et al [38] | Single-blinded randomized  clinical trial | China | EEG | FES | Con-rehab+BCI-FES  n=8 | Con-rehab  n=8 | 20 min/d，5 d/wk,  2 wks, 20 sessions | 1.FMA-UE | - |
| Cheng et al [39] | single-blinded randomized  clinical trial | Singapore | EEG | exoskeleton | Con-rehab+BCI-exoskeleton  n=5 | Con-rehab+exoskeleton  n=6 | 120 min/d, 3 d/wk,  6 wks, 18 sessions | 1.ARAT | 18weeks |
| Kim et al [40] | Double-blinded randomized controlled trial | Korea | EEG | FES | Con-rehab+BCI-FES  n=12 | Con-rehab+FES  n=13 | 90 min/d，5 d/wk,  4 wks, 20 sessions | 1.FMA-UE | - |
| Ang et al [11] | Single-blind randomized controlled trial | Singapore | EEG | exoskeleton | BCI-exoskeleton  n=11 | Exoskeleton  n=14 | 60 min/d，3 d/wk,  4 wks, 12 sessions | 1.FMA-UE | 8weeks |
| Ramos-Murguialday et al [41] | Double-blind randomized controlled trial | Germany | EEG | exoskeleton | BCI-exoskeleton  n=16 | Exoskeleton  n=16 | 60 min/d，5 d/wk,  4 wks, 20 sessions | 1.FMA-UE  2.MAS  3.MAL | - |
| Ramos-Murguialday et al [42] | Double-blind randomized controlled trial | Germany | EEG | exoskeleton | BCI-exoskeleton  n=16 | Exoskeleton  n=12 | 60 min/d，5 d/wk,  4 wks, 20 sessions | 1.FMA-UE  2.MAS  3.MAL | 6months |
| Curado et al [43] | Single-blind randomized controlled trial | Germany | EEG | exoskeleton | BCI-exoskeleton  n=16 | Exoskeleton  n=16 | 60 min/d，5 d/wk,  4 wks, 20 sessions | 1.FMA-UE | - |
| Li et al [44] | Single-blind randomized controlled trial | China | EEG | exoskeleton | BCI-exoskeleton  n=10 | ShamBCI-exoskeleton  n=10 | 60 min/d，5 d/wk,  4 wks, 20 sessions | 1.FMA-UE | - |
| Ma et al [35] | single-blind randomized controlled trial | china | EEG | visual feedback | BCI-MI+Con-rehab  n=20 | Con-rehab  n=20 | 30 min/d，5 d/wk,  4 wks, 20 sessions | 1.FMA-UE | - |
| Mihara et al [45] | Single-blinded randomized controlled trial | Japan | fNIRS | visual feedback | BCI-MI+Con-rehab  n=10 | shamBCI-MI+Con-rehab  n=10 | 20 min/d，3 d/wk,  2 wks, 6 sessions | 1.ARAT  2.FMA-UE  3.MAL | 2weeks |
| Sanders et al [46] | Double-blind randomized controlled trial | UK | fMRI | visual feedback | BCI-MI  n=12 | shamBCI-MI  n=12 | 20 min/d，3 t/d,  3 days, 9 sessions | 1.ARAT  2.FMA-UE | 1month |
| Biasiucci et al [19] | Double-blind randomized controlled trial | Switzerland | EEG | FES | Con-rehab+BCI-FES  n=14 | Con-rehab+shamBCI-FES  n=13 | 60 min/d，2 d/wk,  5 wks, 10 sessions | 1.FMA-UE  2.MAS | 12months |
| Guo et al [47] | single-blind randomized controlled trial | china | EEG | exoskeleton | BCI-exoskeleton  n=10 | Exoskeleton  n=10 | 60 min/d，5 d/wk,  2 wks, 10 sessions | 1.FMA-UE  2.MAS | 3months |
| Kim et al [48] | Single-blind randomized controlled trial | Korea | EEG | FES | BCI-FES+AOT+Con-rehab  n=15 | Con-rehab  n=15 | 30 min/d，5 d/wk,  4 wks, 20 sessions | 1.FMA-UE  2.MAL  3.MBI | - |
| Lee et al [49] | Single-blind randomized controlled trial | China | EEG | FES | BCI-FES+AOT+Con-rehab  n=13 | FES+Con-rehab  n=13 | 30 min/d，5 d/wk,  4 wks, 20 sessions | 1.FMA-UE  2.MAL  3.MBI | - |
| Frolov et al [50] | Single-blind randomized controlled multicenter trial | Russia | EEG | exoskeleton | BCI-exoskeleton  n=30 | Exoskeleton  n=10 | 30 min/d，5 d/wk,  2 wks, 10 sessions | 1.FMA-UE  2.ARAT | - |
| Hu et al [51] | single-blind randomized controlled trial | china | EEG | visual feedback | BCI-MI  n=7 | MI  n=5 | 30 min/d，5 d/wk,  4 wks, 20 sessions | 1.FMA-UE  2.ARAT  3.MBI | - |
| Hao et al [52] | single-blind randomized controlled trial | china | EEG | FES | BCI-FES  n=33 | Con-rehab  n=33 | 20 min/d，7 d/wk,  10 wks, 70 sessions | 1.MAS  2. BBS  3.MBI | - |
| Jinshu et al [53] | single-blind randomized controlled trial | china | EEG | exoskeleton | Con-rehab+BCI-exoskeleton  n=50 | Con-rehab  n=50 | 60 min/d，6 d/wk,  9 wks, 54 sessions | 1.MBI | - |
| Ying et al [54] | single-blind randomized controlled trial | china | EEG | FES | Con-rehab+BCI-FES  n=16 | FES+Con-rehab  n=16 | 30 min/d，3 d/wk,  8 wks, 24 sessions | 1.FMA-UE  2.MBI | - |
| Wang et al [55] | single-blind randomized controlled trial | china | EEG | exoskeleton | AO+BCI-exoskeleton  n=13 | Exoskeleton  n=11 | 30 min/d，4 d/wk,  5 wks, 20 sessions | 1.FMA-UE | 6months |
| EG:Experimental group, CG:Control group, EEG:Electroencephalogram, con-rehab:Conventional rehabilitation, fNIRS:Functional Near-Infrared Spectroscopy, fMRI:Functional Magnetic Resonance Imaging, MI:Motor Imagery, AO:Action Observation | | | | | | | | | |

11. Ang KK, Chua KS, Phua KS, Wang C, Chin ZY, Kuah CW, et al. A Randomized Controlled Trial of EEG-Based Motor Imagery Brain-Computer Interface Robotic Rehabilitation for Stroke. Clinical EEG and neuroscience. 2015 Oct;46(4):310-20. PMID: 24756025. doi: 10.1177/1550059414522229.

19. Biasiucci A, Leeb R, Iturrate I, Perdikis S, Al-Khodairy A, Corbet T, et al. Brain-actuated functional electrical stimulation elicits lasting arm motor recovery after stroke. Nature communications. 2018 Jun 20;9(1):2421. PMID: 29925890. doi: 10.1038/s41467-018-04673-z.

35. Ma ZZ, Wu JJ, Cao Z, Hua XY, Zheng MX, Xing XX, et al. Motor imagery-based brain-computer interface rehabilitation programs enhance upper extremity performance and cortical activation in stroke patients. Journal of neuroengineering and rehabilitation. 2024 May 29;21(1):91. PMID: 38812014. doi: 10.1186/s12984-024-01387-w.

38. Miao Y, Chen S, Zhang X, Jin J, Xu R, Daly I, et al. BCI-Based Rehabilitation on the Stroke in Sequela Stage. Neural plasticity. 2020;2020:8882764. PMID: 33414824. doi: 10.1155/2020/8882764.

39. Cheng N, Phua KS, Lai HS, Tam PK, Tang KY, Cheng KK, et al. Brain-Computer Interface-Based Soft Robotic Glove Rehabilitation for Stroke. IEEE transactions on bio-medical engineering. 2020 Dec;67(12):3339-51. PMID: 32248089. doi: 10.1109/tbme.2020.2984003.

40. Kim MS, Park H, Kwon I, An KO, Kim H, Park G, et al. Efficacy of brain-computer interface training with motor imagery-contingent feedback in improving upper limb function and neuroplasticity among persons with chronic stroke: a double-blinded, parallel-group, randomized controlled trial. Journal of neuroengineering and rehabilitation. 2025 Jan 6;22(1):1. PMID: 39757218. doi: 10.1186/s12984-024-01535-2.

41. Ramos-Murguialday A, Broetz D, Rea M, Läer L, Yilmaz O, Brasil FL, et al. Brain-machine interface in chronic stroke rehabilitation: a controlled study. Annals of neurology. 2013 Jul;74(1):100-8. PMID: 23494615. doi: 10.1002/ana.23879.

42. Ramos-Murguialday A, Curado MR, Broetz D, Yilmaz Ö, Brasil FL, Liberati G, et al. Brain-Machine Interface in Chronic Stroke: Randomized Trial Long-Term Follow-up. Neurorehabilitation and neural repair. 2019 Mar;33(3):188-98. PMID: 30722727. doi: 10.1177/1545968319827573.

43. Curado MR, Cossio EG, Broetz D, Agostini M, Cho W, Brasil FL, et al. Residual Upper Arm Motor Function Primes Innervation of Paretic Forearm Muscles in Chronic Stroke after Brain-Machine Interface (BMI) Training. PloS one. 2015;10(10):e0140161. PMID: 26495971. doi: 10.1371/journal.pone.0140161.

44. Li X, Wang J, Cao X, Huang W, Hu Y, editors. Soft Robotic Glove with Alpha Band Brain Computer Interface for Post-Stroke Hand Function Rehabilitation. 2022 14th Biomedical Engineering International Conference (BMEiCON); 2022: IEEE.

45. Mihara M, Hattori N, Hatakenaka M, Yagura H, Kawano T, Hino T, et al. Near-infrared spectroscopy-mediated neurofeedback enhances efficacy of motor imagery-based training in poststroke victims: a pilot study. Stroke. 2013 Apr;44(4):1091-8. PMID: 23404723. doi: 10.1161/strokeaha.111.674507.

46. Sanders ZB, Fleming MK, Smejka T, Marzolla MC, Zich C, Rieger SW, et al. Self-modulation of motor cortex activity after stroke: a randomized controlled trial. Brain : a journal of neurology. 2022 Oct 21;145(10):3391-404. PMID: 35960166. doi: 10.1093/brain/awac239.

47. Guo N, Wang X, Duanmu D, Huang X, Li X, Fan Y, et al. SSVEP-Based Brain Computer Interface Controlled Soft Robotic Glove for Post-Stroke Hand Function Rehabilitation. IEEE transactions on neural systems and rehabilitation engineering : a publication of the IEEE Engineering in Medicine and Biology Society. 2022;30:1737-44. PMID: 35731756. doi: 10.1109/tnsre.2022.3185262.

48. Kim T, Kim S, Lee B. Effects of Action Observational Training Plus Brain-Computer Interface-Based Functional Electrical Stimulation on Paretic Arm Motor Recovery in Patient with Stroke: A Randomized Controlled Trial. Occupational therapy international. 2016 Mar;23(1):39-47. PMID: 26301519. doi: 10.1002/oti.1403.

49. Lee SH, Kim SS, Lee BH. Action observation training and brain-computer interface controlled functional electrical stimulation enhance upper extremity performance and cortical activation in patients with stroke: a randomized controlled trial. Physiotherapy theory and practice. 2022 Sep;38(9):1126-34. PMID: 33026895. doi: 10.1080/09593985.2020.1831114.

50. Frolov AA, Mokienko O, Lyukmanov R, Biryukova E, Kotov S, Turbina L, et al. Post-stroke Rehabilitation Training with a Motor-Imagery-Based Brain-Computer Interface (BCI)-Controlled Hand Exoskeleton: A Randomized Controlled Multicenter Trial. Frontiers in neuroscience. 2017;11:400. PMID: 28775677. doi: 10.3389/fnins.2017.00400.

51. Hu YQ, Gao TH, Li J, Tao JC, Bai YL, Lu RR. Motor Imagery-Based Brain-Computer Interface Combined with Multimodal Feedback to Promote Upper Limb Motor Function after Stroke: A Preliminary Study. Evidence-based complementary and alternative medicine : eCAM. 2021;2021:1116126. PMID: 34777531. doi: 10.1155/2021/1116126.

52. Hao M, Fang Q, Wu B, Liu L, Tang H, Tian F, et al. Rehabilitation effect of intelligent rehabilitation training system on hemiplegic limb spasms after stroke. Open life sciences. 2023;18(1):20220724. PMID: 37791058. doi: 10.1515/biol-2022-0724.

53. Jinshu Z, Mingming W, Yuan Z, Xuanxiang S. Clinical Research on Rehabilitation Treatment of Hemiplegia after Stroke by Rehabilitation Robot Based on Brain-Computer Interaction Technology. J External Ther Tradit Chin Med. 2021;30(3):3-5. doi: 10.3969/j.issn.1006-978X.2021.03.001.

54. Ying X, Yanyun J, Jie J, Xiaomei W. Efficacy Observation of Brain - Computer Interface Combined with Functional Electrical Stimulation Training on Upper Limb Function and Cognition in Elderly Stroke Patients. Chin J Geriatr Heart Brain Vessel Dis. 2018;20(9):988-90. doi: 10.3969/j.issn.1009-0126.2018.09.023.

55. Wang X, Wong WW, Sun R, Chu WC, Tong KY. Differentiated Effects of Robot Hand Training With and Without Neural Guidance on Neuroplasticity Patterns in Chronic Stroke. Frontiers in neurology. 2018;9:810. PMID: 30349505. doi: 10.3389/fneur.2018.00810.
